# Antigangrenous Serotherapy by a Multivalent Serum

**Published:** 1918-12

**Authors:** 


### *Antigangrenous Serotherapy by a Multivalent Serum.* (second notice.) By MM. H. Vincent and G. Stodel. *C. R. de l'Académie des Sciences*, Aug. 5, 1918.

The authors say that in a recent communication they pointed out that they can prepare an efficacious serum, preventive and curative against gaseous gangrene [1]. The principles according to which they prepared this serum are as follows :

1. H. Vincent et G. Stodel. Sur un sérum préventif et curatif de la gangrène gazeuse. *C. R. de l'Académie des Sciences*, t. 167, p. 137, 16 juillet 1918.

1. The sowing of the tissues taken from a gas gangrene focus reveals a rather large diversity of pathogenous agents. It is actually shown that several anaerobian species are held responsible in the etiology of gaseous gangrene : B. perfringens, chief among them; Vibrion septique; B. oedematiens (Weinberg and Seguin). With these as partners or satellites other anaerobic species may be identified : B. sporogenes, B. putrificus, B. fallax and B. aerofoetidus (Weinberg et Seguin) etc... Sacquépée has frequently found a species he has described under the name of B. bellonensis.

Gaseous gangrene is therefore not simple ; it is a syndrome put in action by various anaerobies, sometimes alone, sometimes in partnership.

Therefore, in order to be efficacious and really specific, serotherapy of gaseous gangrene must simultaneously aim at the different microbes capable of causing the infection. Otherwise it merely gives negative or insufficient results.

2. The lesions caused in the course of the infection arise :

a) from the fact that the tissues are invaded by the anaerobic bacilli (microbian action) ; b) from poisoning of the living cells (muscular and cellular tissues, hematiae), likewise of the nervous system, by the secreted products of those microbes (proteolytic and toxic action).

*That is the reason why a serum must be at the same time antimicrobian and antitoxic in order to be efficacious.*

3. Former experiments[1] have shown the importance of microbian associations in the multiplication of certain anaerobies. The searches for Vibrion septique by Besson, those by Weinberg, and those we have made ourselves, show that the association of the pathogenic species (C. perfringens, V. septique, B. oedematiens, etc.) is much more virulent and more rapidly mortal than the same dose of each of those bacilli injected separately. The intramuscular injection of three of those microbes (B. perfringens, Vibrion septique, B. sporogenes) kills a guinea pig of 400 grammes in 10 to 18 hours at the dose 1/10 cc. It is not, therefore, by mixing sera taken from horses immunized separately against each of the bacilli causing gaseous gangrene that a serum giving the utmost protective and curative action can be obtained. That optimum result will be realized by injecting the same horse with the combination of those pathogenous cultures, as their association increases their virulence considerably.

For the preparation of our serum, we use fourteen species or

1. Vaillard and H. Vincent. Contribution à l'étude du tétanos. *C. R. de l'Académie des Sciences*, Janvier 1891, and *Annales de l'Institut Pasteur*, 25 Octobre 1896.

races including B. perfringens, Vibrion septique, B. oedematiens, B. sporogenes, B. putrificus, B. bellonensis[1], etc...

We have controlled their pathogenic influence by its inoculation in the guinea-pig. Finally, each time that the sowing of gangrenous tissues from man supplies us with a particularly active strain, this organism is added to those used by us for the immunization of our horses.

They began simultaneously immunizing these same horses against tetanus.

Scientists who have studied the serotherapy of gaseous gangrene have not, thus far, made known their modus operandi.

They have, however, characterized the method as "antigene". They employ total cultures in simple broth, or in glucosed broth at 2 per 1,000, or else the "centifrugat" of those cultures.

By this means, and also by the use of cultures on a solid medium, the authors have obtained sera, the activity of which appeared insufficient or less complete, among animals subjected to the special test control already described[2].

Besides, the injection of large quantities of cultures in the vein of the horse produces serious complications by reason of the foreign substances which these cultures introduce into the blood.

Such injections produce serious morbid symptoms (oedema, arthritis, acute thinness) frequently necessitating the interruption of the immunization.

That is why we have employed another method[3].

Each pathogenous microbe is cultivated on agar. Emulsions are obtained from it in physiological solution. The bottles containing these microbian suspensions, under a layer of oil, are placed in the autoclave heated to 38°. They are left there from 2 to 4 days or even more. This second culture becomes more abundant and gives rise to odoriferous gas; this medium abounds in endo and in exotoxines.

These mixed macerations are injected in the initial dose of 10 c.c., then in progressive doses, in the veins of horses. These can provide a most protecting serum after the third month. Experiments are being made to shorten the duration of immunization by the initial injection of anti-gangrenous serum and "antigene".

The serum thus obtained has realized unexpected cures. Since our preceding communication, it has realized cures even of severe

1. We thank M. Sacquépée who kindly sent us a sample of his bacillus.
2. H. Vincent et G. Stodel, *C. R. de l'Académie des Sciences*, 1917, p. 870, and 1918, p. 137.
3. This method can be applied to the preparation of other serums (H. Vincent)

forms, and — an important fact — it has saved the limbs of several wounded men. We will make these results known.

